# Trial to re-evaluate ultrasound in the treatment of tibial fractures (TRUST): a multicenter randomized pilot study

**DOI:** 10.1186/1745-6215-15-206

**Published:** 2014-06-04

**Authors:** Jason W Busse, Mohit Bhandari, Thomas A Einhorn, James D Heckman, Kwok-Sui Leung, Emil Schemitsch, Paul Tornetta, Stephen D Walter, Gordon H Guyatt

**Affiliations:** 1 Department of Anesthesia, McMaster University, HSC-2U1, 1200 Main St. West, Hamilton, ON L8S 4 K1, Canada; 2 Department of Biostatistics and Clinical Epidemiology, McMaster University, 1280 Main Street West, Hamilton, ON L8S 4 K1, Canada; 3 The Michael G DeGroote Institute for Pain Research and Care, McMaster University, 1280 Main Street West, Hamilton, ON L8S 4 K1, Canada; 4 Department of Surgery, McMaster University, 1280 Main Street West, Hamilton, ON L8S 4 L8, Canada; 5 Department of Orthopedic Surgery, Boston University Medical Center, 725 Albany Street, Shapiro, Suite 4B, Boston, MA 02118, USA; 6 Department of Orthopedic Surgery, Dartmouth-Hitchcock Medical Center, One Medical Center Drive, Lebanon, NH 03756, USA; 7 Department of Orthopedics and Traumatology, the Chinese University of Hong Kong, Professorial Block, Queen Mary Hospital, Pok Fu Lam, Hong Kong, China; 8 Division of Orthopedic Surgery, University of Toronto, 149 College Street, Toronto, ON M5T 1P5, Canada; 9 Department of Medicine, McMaster University, 1280 Main Street West, Hamilton, ON L8S 4 K1, Canada

**Keywords:** Ultrasound, Fracture healing, Randomized controlled trial

## Abstract

**Background:**

The role of low-intensity pulsed ultrasound (LIPUS) in the management of fractures remains controversial. The purpose of this study was to assess the feasibility of a definitive trial to determine the effect of LIPUS on functional and clinical outcomes in tibial fractures managed operatively.

**Methods:**

We conducted a multicenter, concealed, blinded randomized trial of 51 skeletally mature adults with operatively managed tibial fractures who were treated with either LIPUS or a sham device. All participating centers were located in Canada and site investigators were orthopedic surgeons specializing in trauma surgery. The goals of our pilot study were to determine recruitment rates in individual centers, investigators’ ability to adhere to study protocol and data collection procedures, our ability to achieve close to 100% follow-up rates, and the degree to which patients were compliant with treatment. Patients were followed for one year and a committee (blinded to allocation) adjudicated all outcomes. The committee adjudicators were experienced (10 or more years in practice) orthopedic surgeons with formal research training, specializing in trauma surgery.

**Results:**

Our overall rate of recruitment was approximately 0.8 patients per center per month and site investigators successfully adhered to the study protocol and procedures. Our rate of follow-up at one year was 84%. Patient compliance, measured by an internal timer in the study devices, revealed that 39 (76%) of the patients were fully compliant and 12 (24%) demonstrated a greater than 50% compliance. Based on patient feedback regarding excessive questionnaire burden, we conducted an analysis using data from another tibial fracture trial that revealed the Short Musculoskeletal Function Assessment (SMFA) dysfunction index offered no important advantages over the SF-36 Physical Component Summary (PCS) score. No device-related adverse events were reported.

**Conclusions:**

Our pilot study identified key issues that might have rendered a definitive trial unfeasible. By modifying our protocol to address these challenges we have enhanced the feasibility of a definitive trial to explore the effect of LIPUS on tibial fracture healing.

**Trial registration:**

The TRUST definitive trial was registered at ClinicalTrials.gov on 21 April 2008 (identifier: NCT00667849).

## Background

Tibial fractures, one of the most common long-bone fractures [1], typically require three to six months before patients are experiencing only minimal pain and have returned to their pre-injury functional status. This injury is associated with substantial loss in productivity and, moreover, tibial fractures are prone to complications [2-5]. The limited soft tissue envelope surrounding the bone predisposes tibial fractures to fail to unite (non-unions), a complication affecting approximately 10% of tibial fracture patients in North America each year [6]. Non-unions require surgery to promote fracture healing, surgery that is associated with its own complications.

One management strategy currently in use to minimize both fracture healing time and complication rates is low-intensity pulsed ultrasound (LIPUS). A quarter of respondents to a survey of Canadian orthopedic surgeons and senior physiotherapy students (n = 77; 77% response rate) reported use of LIPUS, although 21% of senior physiotherapy students opined that LIPUS was contraindicated for fracture healing due to concerns of damaging healing bone and 25% of surgeons opined that LIPUS had no effect on bone healing [7]. Of 268 respondents to a survey of 450 surgeon members of the Canadian Orthopedic Association (60% response rate), 45% reported use of bone stimulators for fracture healing in at least some cases, evenly split between LIPUS and electrical stimulation [8].

Although a number of randomized trials have suggested that LIPUS may improve fracture healing, inferences are limited because of small sample size, risk of bias, frequent reporting of surrogate outcomes (such as radiographic healing) but limited attention to patient-important outcomes (functional recovery), and inconsistent results [9]. Until a large randomized controlled trial (RCT) is undertaken, the effect of LIPUS on fracture healing will remain uncertain. The purpose of the current pilot study was to explore the feasibility of a definitive trial to establish the role of LIPUS for tibial fracture healing, specifically: to determine recruitment rates in individual centers, adherence to study protocol and data collection procedures, our ability to achieve close to 100% follow-up rates, and the degree to which patients complied with treatment.

### Availability of data

For this research, full access to the data was not available. However, the Editors-in-Chief considered it important to publish the article to help disseminate the information that was available.

## Methods

### Trial design

We originally set out to recruit non-operatively managed tibial fracture patients. After four months we had not enrolled a single patient. A survey of 450 Canadian trauma surgeons (60% response rate) revealed a shift in practice away from non-operative to operative management of tibial fractures in the four years since we had collected the data on which we based initial recruitment estimates [8]. We therefore revised our eligibility criteria to focus on operatively managed tibial fractures.

Between March 2006 and June 2007 six Canadian trauma centers recruited 51 patients. All participating centers were university-affiliated academic trauma hospitals. Site principal investigators were trauma trained orthopedic surgeons. The human subjects committees (REB#05-171 - Research Ethics Boards/Institutional Review Boards) approved the standardized protocol at each participating site. Specifically, the Hamilton Health Sciences/McMaster University Research Ethics Board, the University of Western Ontario Research Ethics Board, the Ottawa Hospital Research Ethics Board, St. Michael's Hospital Research Ethics Board, and Sunnybrook Health Sciences Center Research Ethics Board. We developed, but did not register, a protocol prior to conduct of the pilot study.

Visually identical active and inactivated (sham) EXOGEN 2000+ ultrasound device units with a unit number were labeled and shipped from the manufacturer (Smith & Nephew, Memphis, TN) to investigational sites according to the randomization plan established by the Methods and Coordinating Center (CLARITY Methods Center at McMaster University). The CLARITY Methods Center at McMaster University was responsible for the traceability of all investigational devices under this trial.

Participating investigators randomized patients by accessing a 24-hour toll-free remote telephone randomization system that ensured concealment. Randomization was stratified by center and by severity of soft-tissue injury (open or closed) in randomly permuted blocks. Patients and clinicians were unaware of block sizes. After fracture fixation with an intramedullary nail, patients were allocated by a local research coordinator (in a 1:1 ratio) to LIPUS or a deactivated LIPUS device. The active and placebo-treatment devices were identical in every way with the exception of the administration of ultrasound, in that they had the same visual, tactile, and auditory signals. To ensure similar perioperative regimens, participating centers standardized key aspects of pre- and post-operative care. Treating clinicians were all trauma trained orthopedic surgeons. Patients, surgeons, and other clinicians, data collectors, outcome adjudicators, and data analysts were blinded to treatment allocation until the data analysis was complete (analysis was done as arms A and B only). We collected outcome data on patients at discharge and at 6 weeks, 3, 4, 5, 6, 9, and 12 months postoperatively.

### Eligibility criteria

Eligible patients were skeletally mature (age 18 years or older) men or women with an open (Gustillo Grade I-IIIB) or closed (Tscherne Grade 0-3) tibial fracture amenable to intramedullary nail fixation. For open fractures, debridement had to have taken place within 24 hours of presentation. Inclusion required written informed consent for trial participation, initiation of treatment with a LIPUS device within 14 days of intramedullary nailing, and verbal commitment to comply with the study protocol and return for all follow-up evaluations.

We excluded patients with: circumferential, open wounds that precluded placement of an ultrasound device at the fracture site, general wound care that precluded ultrasound-skin contact, pilon fractures, tibial fractures that extended into the knee or ankle joint and required reduction, pathologic fractures, bilateral tibial fractures, segmental fractures, spiral fractures more than 3 inches in length, concomitant injuries which, in the opinion of the attending surgeon, were likely to impair function for at least as long as the patient’s tibial fracture, or tibial fractures that showed less than 25% cortical contact and more than a 1 cm gap following intramedullary nail fixation. We also excluded women who were pregnant or nursing or who planned to become pregnant over the course of treatment, patients with active implantable devices such as cardiac pacemakers, those with cognitive impairment or language difficulties that might impede the valid completion of questionnaires, and those who were likely to have problems with maintaining follow-up. The central adjudication committee (CAC), including the co-principal investigator (MB, orthopedic surgeon/methodologist), and two orthopedic surgeons (DS and EHS) adjudicated eligibility.

### Interventions

Each patient received a LIPUS device (the EXOGEN 2000+) manufactured by Smith & Nephew, verbal instructions on its use, and a booklet containing detailed instructions. Neither the patient nor the clinical investigator was able to adjust the ultrasound signal. In brief, after the application of a small amount of ultrasonic coupling gel to the surface of the ultrasound head, the patient positioned the treatment head module over the fracture site and turned on the LIPUS device which automatically turns off after 20 minutes. The active and placebo-treatment devices were identical except for the administration of ultrasound.

Patients self-administered treatment with their device once daily. Treatment continued until the CAC determined that the fracture demonstrated radiographic evidence of bridging at all four cortices, or until the 52-week follow-up visit, whichever occurred first. The EXOGEN 2000+ contains an internal patient use timer which monitors and records use. When the device turns on, it initially performs a self-test after which it displays the total number of full treatments completed, then the number of partial treatments. If the device is removed prior to completion of the daily treatment it shuts off and records the abbreviated treatment time. Patient compliance with ultrasound treatment was ascertained using the patient compliance monitor function of the device at each follow-up office visit until their fracture had healed (defined as cortical bridging at three of the four cortices). The compliance monitor, however, cannot establish if the device had been used properly.

### Standardization of perioperative treatment

For closed fractures preoperative antibiotic administration was continued for 24 hours postoperatively. For open fractures preoperative intravenous antibiotic administration included a cephalosporin and an aminoglycoside that were continued for 72 hours postoperatively. Irrigation and debridement of soft tissues and contaminated bone was repeated as necessary and delayed wound closure, split thickness skin grafting, or muscle flaps (for grade IIIB only) occurred only after the initial surgery. For both open and closed fractures cortical contact of the fracture ends guided weight bearing. If cortical contact was achieved patients were instructed to weight bear as tolerated. Otherwise, patients were instructed to partially weight bear on the affected limb until performance of a definitive procedure to achieve contact.

### Outcome measures

The goals of our pilot study were to determine recruitment rates in individual centers, adherence to study protocol and data collection procedures, our ability to achieve close to 100% follow-up rates, and the degree to which patients complied with treatment.

The primary effectiveness outcome measure was the Physical Component Summary (PCS) score of the SF-36. The SF-36 is a widely accepted, well-validated functional status measure [10,11]. It is a self-administered, 36-item questionnaire that measures health-related quality of life in eight domains. Each domain is scored from 0 (lowest level) to 100 (highest level). Physical and mental summary scores can be obtained by aggregating across domains. The SF-36 has demonstrated good construct validity, high internal consistency, and high test-retest reliability [12]. In a previous study, we have shown that three out of four SF-36 PCS domains are responsive to improvement in functional recovery in patients with ankle fractures over a period of one year [13]. The primary safety outcome was the difference between treatment groups in the proportion of patients with device-related adverse events and unplanned secondary procedures related to bone healing and infection.

Secondary outcomes were radiographic healing, rates of malunion and nonunion, rates of secondary procedures (operative and non-operative), the Short Musculoskeletal Function Assessment (SMFA) dysfunction index, and the Health Utilities Index-III (HUI-III) (an extensively validated generic utility measure [14-16]). The SMFA is a two part, 46-item, self-reported health status questionnaire [17]. One part (the dysfunction index) is designed to detect changes in the functional status of patients who have a broad range of musculoskeletal disorders that are commonly seen in community practices.

We defined a malunion as an angular or rotational deformity in either the coronal (5 degrees) or sagittal plane (10 degrees). A non-union was defined as a failure of progression of radiographic healing in two consecutive months (after the six-month postoperative visit). The Radiographic Union Scale for Tibial Fractures (RUST) system assigns a score to a given set of anteroposterior and lateral radiographs based on the assessment of healing at each of the four cortices visible on these projections (medial and lateral cortices on the anteroposterior X-ray, anterior and posterior cortices on the lateral X-ray) [18-20]. Each cortex receives a score of one point if it is deemed to have a fracture line with no callus, two points if there is callus present but a fracture line is still visible, and three points if there is bridging callus with no evidence of a fracture line. The individual cortical scores are added to give a total for the set of films, with four being the minimum (indicating the fracture has definitely not healed) and twelve being the maximum score (indicating the fracture has definitely healed). Our CAC judged that a cortex was bridged when it achieved a RUST score of two or three. Anteroposterior and lateral radiographs were standardized whenever possible, with use of the same X-ray machine at each site and the same exposure settings.

### Follow-up

We assessed all outcomes at discharge and at follow-up visits, with the exception of radiographic healing which was evaluated until the CAC determined that the bridging of three cortices had occurred. The CAC, which was blinded to device allocation, adjudicated all outcomes and resolved disagreement through discussion. All centers sent digital photographs of the required radiographs to the TRUST Methods Center via email. In addition, site coordinators mailed all relevant hospital records. All relevant blinded patient records (DataFax, Hamilton, Ontario, Canada; case report forms, chart notes, and radiographs) were posted on a specially designed, password-protected, website for adjudication. Disagreements were resolved by discussion during conference calls. If the adjudicators could not reach consensus, additional information was requested from the participating site to clarify areas of uncertainty. All decisions made by the committee were final.

### Sample size

We estimated that 500 patients would be required for a definitive trial to have a power of 80% (alpha = 0.05, two-tail) to identify a patient-important difference in absolute SF-36 PCS scores (3 to 5 points) [21] between treatment and control groups, across a plausible range in the magnitude of the standard deviation (12 to 14) [13]. We chose a sample size of 50 patients to assess feasibility. No interim analyses were planned.

### Statistical analyses

Although not the primary objective of our pilot study, we conducted preliminary repeated measures of variance analyses, adjusted for treatment, time, fractures-at-risk, baseline questionnaire scores (only for SF-36 PCS and HUI-III scores), and three interaction terms: treatment × time, treatment × fractures-at-risk, and time × fractures-at-risk. Our analyses followed the intention-to-treat principle and all patients were analyzed according to the group they were randomized to. We defined patients with fractures-at-risk *a priori* as those presenting with any of the following at enrolment: fracture gap (all enrolled fracture gaps were less than or equal to 1 cm), current smoker, or open fracture. We set our level of statistical significance at *P* <0.05. Our analyses were restricted to those patients who provided completed questionnaires or radiographs (complete case analysis). When follow-up time differed from that specified in the protocol, we used follow-up to the scheduled time point closest to the actual follow-up. If two follow-up times were equally near to a single scheduled time point, we used data from the earlier follow-up time.

### Funding and role of the sponsor

The Trial to Re-evaluate Ultrasound in the Treatment of Tibial Fractures (TRUST) was funded under an industry-partnered research grant from the Canadian Institutes of Health Research and Smith & Nephew. The Canadian Institutes of Health Research had no role in the design or conduct of the study, the collection, analysis, and interpretation of the study, or the preparation, review, or approval of the manuscript. Smith & Nephew personnel reviewed initial drafts of the trial protocol and raised many issues about alternative approaches to study design. Issues regarding the protocol were resolved through negotiation between Smith & Nephew and the trial Steering Committee. Final decisions regarding the protocol and issues that might arise during the conduct of the trial were the purview of the trial Steering Committee. The investigators collected all trial data, with the exception of patient use of ultrasound devices which was recorded by an internal timer in each device. A summary of these data were obtained from Smith & Nephew when study devices were returned to them (categorized as fully complaint or partially complaint); the investigators were not provided with the actual time of usage per patient. Smith & Nephew had no role in the initial preparation of the current study manuscript but had the right to review the manuscript and make nonbinding comments and suggestions.

## Results

**Figure 1:**
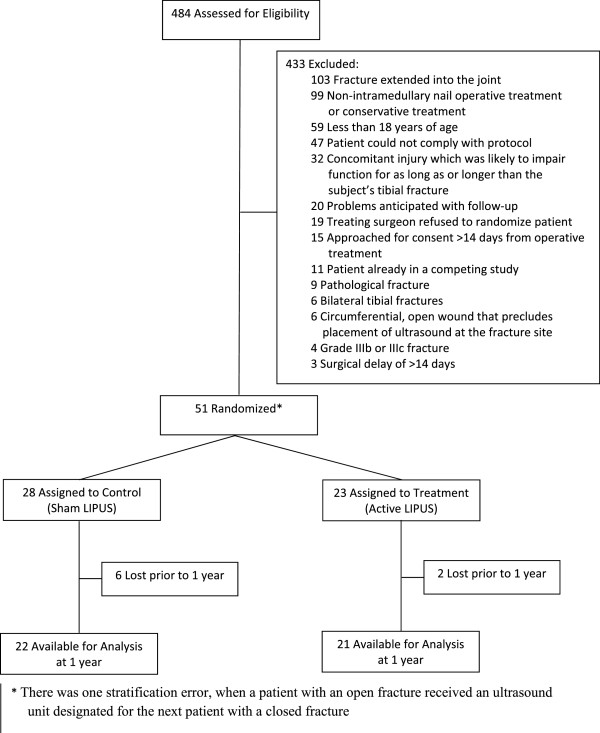
Patient recruitment and follow-up schedule (CONSORT flow diagram).

Of the 484 patients who were screened for eligibility, 51 met our study criteria, provided informed consent and were randomized, for an overall recruitment rate of 10.5%; 37 were closed fractures and 14 open fractures (Figure 1). Patient enrollment by center was as follows: Hamilton Health Sciences, Hamilton (n = 22); London Health Sciences Center, London (n = 12), Queen Elizabeth II Health Sciences Center, Halifax (n = 7); St. Michael’s Hospital, Toronto (n = 6); Sunnybrook Hospital, Toronto (n = 2); and Ottawa Hospital, Ottawa (n = 2).

According to our definitions, we enrolled 28 patients at high risk of poor outcome and 23 patients at low risk. Our overall rate of recruitment was approximately 0.8 patients per center, per month; this rate varied by site from 0.2 to 2 patients per month. We reviewed our 433 ineligible patients and found that many (23%) were excluded because their tibial shaft fracture extended into the joint (Figure 1). Patients were predominantly males who had been injured in a fall (Table 1).

**Table 1:** Patient characteristics

	**Sham device**	**LIPUS**	**Total**
	**(n = 28)**	**(n = 23)**	**(n = 51)**
Age (years), mean (SD)	39.6 (13.6)	39.0 (13.6)	39.3 (13.5)
Females, n(%)	7 (25.0)	5 (21.7)	12 (23.5)
Mechanism of injury, n(%)			
MVA, driver or passenger	4 (14.3)	1 (4.3)	5 (9.8)
MVA, pedestrian	1 (3.6)	1 (4.3)	2 (3.9)
Motorcycle accident	3 (10.7)	4 (17.4)	7 (13.7)
Crush injury	3 (10.7)	4 (17.4)	7 (13.7)
Fall	10 (35.7)	9 (39.1)	19 (37.3)
Twist	1 (3.6)	1 (4.3)	2 (3.9)
Direct trauma, blunt	2 (7.1)	2 (8.7)	4 (7.8)
Recreational vehicle injury	3 (10.7)	1 (4.3)	4 (7.8)
Hockey injury	1 (3.6)	0 (0.0)	1 (2.0)
Diabetic, n(%)	1 (3.6)	0 (0.0)	1 (2.0)
Current smoker, n(%)	10 (35.7)	6 (26.1)	16 (31.4)
Type of fracture, n(%)			
Open	9 (32.1)	5 (21.7)	14 (27.5)
Closed	19 (67.9)	18 (78.3)	37 (72.5)
AO Class, n(%)			
A	13 (46.4)	11 (47.8)	24 (47.1)
B	9 (32.1)	6 (26.1)	15 (29.4)
C	6 (21.4)	6 (26.1)	12 (23.5)
Any comorbidity^1^, n(%)	3 (10.7)	1 (4.3)	4 (7.8)
Post-surgical fracture gap, n(%)			
Any gap (restricted to ≤1 cm)	6 (21.4)	0 (0.0)	6 (11.8)
No gap	22 (78.6)	23 (100.0)	45 (88.2)
Fracture-at-risk^2^, n(%)	21 (75.0)	12 (52.2)	33 (64.7)

^1^diabetes, renal disease, vascular disease or insufficiency, thrombophlebitis, alcoholism, or drug abuse.

^2^open fracture, current smoker, comorbidity, AO class C, or any fracture gap ≤1 cm.

AO: Arbeitsgemeinschaft für Osteosynthesefragen; LIPUS: low-intensity pulsed ultrasound; MVA: motor vehicle accident; SD: standard deviation; TRUST: Trial to Re-evaluate Ultrasound in the Treatment of Tibial Fractures.

We recorded one stratification error during the pilot study when a patient with an open fracture received an ultrasound unit designated for the next patient with a closed fracture, but all other patients received their randomly assigned treatment. Trial sites successfully adhered to protocol and provided full data for all follow-up visits that patients attended, except for inconsistent completion of the SMFA which was attributed to excessive respondent burden.

Patient compliance as measured by the ultrasound device was high, with 39 (76%) registering full compliance and 12 (24%) registering greater than 50% compliance. Nine patients stopped treatment early, two started late, and one patient started late and stopped early. Reasons provided were forgetting the ultrasound unit while traveling, other forgetfulness, or believing that further treatment was unnecessary because of perception that the fracture was healed. One patient initially stopped treatment early due to losing his coupling gel but resumed once replacement gel was provided. One patient reported applying ultrasound to both the fracture site and another site, which was corrected by instruction from his site’s research coordinator.

Of our 51 enrolled patients, 1 withdrew consent after 6 weeks following advice from his physiotherapist that the ultrasound device would damage healing bone, and 1 was lost to follow-up after his fracture was declared healed and he left the country; another 6 patients failed to return for assessment at 1 year for unknown reasons. As such, we had 16% of enrolled patients (8 of 51) lost to follow-up. Overall, we successfully completed 82% of all follow-up visits; follow-up rates varied by site from 43% to 98%.

Repeated measures of variance analysis found that treatment with LIPUS versus sham therapy was not significantly associated with improvement in SF-36 PCS scores (time × treatment interaction, *P* = 0.27), HUI-III scores (time × treatment interaction, *P* = 0.31), or RUST scores (time × treatment interaction, *P* = 0.53). Patient with fractures-at-risk demonstrated worse outcomes across all three measures. The adjusted means for SF-36 PSC, HUI-III, and RUST scores at each time point, and a breakdown by fracture risk status are provided elsewhere (see Additional files 1, 2 and 3).

There were 50 reported adverse events in the pilot study. Eight were reported at the time of enrolment and before the administration of the first treatment. The remaining 42 adverse events included: 6 wound infections (3 in the same patient), 6 hardware removals to alleviate discomfort, 6 irrigations and debridements due to infection (2 in same patient), 4 hardware removals for dynamization, 2 recurrences of pneumonia originally acquired prior to enrolment (in the same patient), 1 hospitalization for deep vein thrombosis and pulmonary embolism, 1 intramedullary nail loosening suggestive of a deep infection, and 1 case of neurapraxia (see Additional file 4). No site investigators reported any adverse events that they believed to be associated with the study treatment.

## Discussion

Our pilot study demonstrated that a definitive trial to establish the effect of LIPUS on tibial fracture healing is feasible. Specifically, we determined recruitment rates in individual centers, found excellent adherence to study protocol and data collection procedures among centers, achieved an acceptable follow-up rate (84%), and established that most patients are compliant with treatment.

Our pilot study revealed highly variable recruitment rates among individual trauma centers. Most patients (89.5%) presenting with tibial fractures that we screened were not eligible for enrolment, often due to fractures extending into the joint. We successfully completed 82% of follow-up visits, and adherence to study protocol and data collection procedures were excellent with the exception of completion of the SMFA. Our analysis of our primary outcome and certain secondary outcome measures was exploratory and an appropriately powered analysis from our definitive trial, which is in progress, will be required to confirm or refute our preliminary findings. Our findings that patients who smoke, those with open tibial fractures, and those with a fracture gap demonstrate worse outcomes as measured by SF-36 PCS, HUI-III, and RUST scores suggest that our definitive trial should consider fractures-at-risk for subgroup analyses.

On consultation, both the manufacturers of the Exogen ultrasound bone stimulator device and our consultant trauma surgeons opined that tibial fractures extending into the joint would respond to LIPUS treatment in a similar way to currently eligible patients. Accordingly, for our definitive trial we have expanded our eligibility criteria to include tibial shaft fractures that extend into the joint and do not require reduction. Given our recruitment data, we concluded that our definitive trial should engage 25 trauma centers for patient enrolment, and that we should set *a priori* recruitment rate criteria for centers to remain actively recruiting in the TRUST trial; specifically, all participating sites must enroll at least one patient every four months.

To explore if the data from the SMFA justified the respondent burden, we analyzed data from the recently completed SPRINT trial [22], which also studied operatively managed tibial shaft fractures (n = 1339) and administered both the SF-36 and SMFA. Both instruments proved responsive, instrument scores were highly correlated (correlation at 1-year post surgery was -0.80 (95% confidence interval = -0.83 to -0.77)), and the difference in standardized change scores between instruments was not significant (standardized response mean of 0.95 versus 1.00, *P* = 0.16) [23]. Given these results, we concluded that the additional information from administering both questionnaires was insufficient to justify the respondent burden. The far more extensive use of the SF-36, its more extensive validation, and its ability to compare to other populations dictates the SF-36 as the instrument of choice.

To further improve patient compliance in the definitive TRUST trial we are providing each TRUST site with extra coupling gel, informing all patients at enrolment that replacement gel is available, and instructing all patients at enrolment that their LIPUS device is to be used only at the indicated fracture site. The ultrasound devices to be used in the definitive TRUST trial have been upgraded to reduce weight and size and thereby improve portability. Site research coordinators will reinforce the importance of compliance with the prescribed treatment administration at each patient follow-up visit.

To improve the rate of follow-up in the definitive TRUST trial, enrolled patients’ physiotherapists will be sent a letter outlining the current evidence about treatment of fractures with LIPUS and the safety of this modality. All site research coordinators and investigators will be informed that patients who leave the country, or migrate within the country, can be followed up using telephone administration of outcome measures. Finally, we will provide financial compensation to patients to cover travel costs for follow-up visits.

## Conclusions

Our pilot study identified key issues that might have rendered a definitive trial unfeasible. By switching to an operatively managed population, broadening our eligibility criteria, reducing the number of study questionnaires, and taking additional steps to encourage higher rates of patient compliance and follow-up, we have enhanced the feasibility of a definitive trial to explore the effect of LIPUS on tibial fracture healing. Our results illustrate the potential and value of pilot studies before embarking on definitive orthopedic trials.

## Abbreviations

CAC: Central adjudication committee; HUI-III: Health Utilities Index-III; LIPUS: Low-intensity pulsed ultrasound; PCS: Physical Component Summary; RCT: Randomized controlled trial; RUST: Radiographic Union Scale for Tibial Fractures; SMFA: Short Musculoskeletal Function Assessment; TRUST: Trial to Re-evaluate Ultrasound in the Treatment of Tibial Fractures.

## Competing interests

Drs Einhorn, Schemitsch, and Bhandari have received consulting fees from Smith & Nephew, the manufacturer of the study device. Dr Tornetta receives royalties from Smith & Nephew.

## Authors’ contributions

JWB, MB, TAE, JDH, K-SL, ES, PTIII, SDW, and GHG conceived of the study and contributed to the design. JWB, DH-A, and GHG analyzed the data. JWB wrote the first draft of the paper. All authors read and approved the final manuscript.

## Supplementary Material

Additional file 1 **Adjusted Mean SF-36 PCS Scores.** Description of data: A comparison of adjusted mean SF-36 PCS scores for the treatment and control groups, at each follow-up time. Click here for file

Additional file 2 **Adjusted Mean HUI-III Scores.** Description of data: A comparison of adjusted mean HUI-III scores for the treatment and control groups, at each follow-up time. Click here for file

Additional file 3 **Adjusted Mean RUST Scores.** Description of data: A comparison of adjusted mean RUST scores for the treatment and control groups, at each follow-up time. Click here for file

Additional file 4 **Adverse events.** Description of data: Adverse events reported in both treatment arms. Click here for file
