# Supplementary material for: Trial to re-evaluate ultrasound in the treatment of tibial fractures (TRUST): a multicenter randomized pilot study
Source: Trials. 2014 Jun 4;15:206. doi: 10.1186/1745-6215-15-206 (PMC4060850; doi:10.1186/1745-6215-15-206)
Supplement: Additional file 1 — Adjusted Mean SF-36 PCS Scores. Description of data: A comparison of adjusted mean SF-36 PCS scores for the treatment and control groups, at each follow-up time. [file 1745-6215-15-206-S1.pdf]

Additional file 1: Adjusted Mean SF-36 PCS Scores [1,2]

	N	Sham Device<br>Mean (95% CI)	LIPUS<br>Mean (95% CI)	Difference<br>Mean (95% CI)
**All patients**				
6-week	50	32.92 (29.26, 36.59)	32.54 (28.51, 36.56)	0.39 (-5.21, 5.98)
3-month	43	36.10 (32.29, 39.90)	36.18 (32.12, 40.25)	-0.09 (-5.82, 5.65)
4-month	42	41.22 (37.32, 45.12)	39.67 (35.61, 43.73)	1.55 (-4.27, 7.37)
5-month	36	42.32 (38.33, 46.30)	42.84 (38.69, 47.00)	-0.52 (-6.47, 5.42)
6-month	40	42.41 (38.45, 46.38)	45.82 (41.65, 50.00)	-3.41 (-9.35, 2.53)
9-month	38	45.22 (41.09, 49.35)	49.31 (45.04, 53.59)	-4.09 (-10.21, 2.03)
12-month	43	47.71 (43.65, 51.78)	46.86 (42.73, 50.99)	0.86 (-5.11, 6.82)
**High Risk Fractures**				
6-week	28	32.35 (28.10, 36.60)	32.76 (27.22, 38.31)	-0.41 (-7.18, 6.36)
3-month	24	33.18 (28.83, 37.53)	34.07 (28.47, 39.67)	-0.89 (-7.74, 5.97)
4-month	25	38.52 (34.16, 42.87)	37.77 (32.15, 43.39)	0.75 (-6.15, 7.65)
5-month	19	39.99 (35.49, 44.49)	41.32 (35.58, 47.05)	-1.33 (-8.33, 5.67)
6-month	22	40.00 (35.51, 44.50)	44.21 (38.48, 49.94)	-4.21 (-11.20, 2.78)
9-month	23	42.20 (37.57, 46.82)	47.09 (41.38, 52.81)	-4.89 (-11.99, 2.20)
12-month	25	43.46 (38.87, 48.05)	43.40 (37.85, 48.96)	0.06 (-6.89, 7.00)
**Low Risk Fractures**				
6-week	22	33.68 (27.90, 39.47)	32.24 (27.30, 37.18)	1.45 (-5.81, 8.70)
3-month	19	39.94 (33.93, 45.96)	38.97 (33.99, 43.96)	0.97 (-6.43, 8.37)
4-month	17	44.78 (38.56, 51.01)	42.18 (37.19, 47.17)	2.61 (-4.89, 10.11)
5-month	17	45.39 (39.11, 51.66)	44.85 (39.81, 49.90)	0.53 (-7.07, 8.13)
6-month	18	45.59 (39.33, 51.85)	47.94 (42.85, 53.04)	-2.35 (-9.96, 5.25)
9-month	15	49.20 (42.67, 55.73)	52.24 (46.81, 57.67)	-3.04 (-10.85, 4.77)
12-month	18	53.32 (46.94, 59.71)	51.41 (46.15, 56.67)	1.91 (-5.79, 9.62)

1. Adjusted for treatment, time, fractures-at-risk, baseline questionnaire score, treatment x time, treatment x fractures-at-risk, and time x fractures-at-risk.

2. Repeated measures of variance analysis found a significant effect of time ($p<0.01$), fractures-at-risk ($p=0.03$), and baseline SF-36 PCS score ($p=0.01$) on SF-36 PCS scores. Our analysis failed to show an effect of treatment x time ($p=0.27$), treatment x fractures-at-risk ($p=0.66$), or time x fractures-at-risk ($p=0.11$) on SF-36 PCS scores.
