# Supplementary material for: Trial to re-evaluate ultrasound in the treatment of tibial fractures (TRUST): a multicenter randomized pilot study
Source: Trials. 2014 Jun 4;15:206. doi: 10.1186/1745-6215-15-206 (PMC4060850; doi:10.1186/1745-6215-15-206)
Supplement: Additional file 2 — Adjusted Mean HUI-III Scores. Description of data: A comparison of adjusted mean HUI-III scores for the treatment and control groups, at each follow-up time. [file 1745-6215-15-206-S2.pdf]

Additional file 2: Adjusted Mean HUI-III Scores [1,2]

	N	Sham Device	LIPUS	Difference
		**Mean (95% CI)**	**Mean (95% CI)**	**Mean (95% CI)**
**All patients**				
6-week	48	0.47 (0.38, 0.57)	0.47 (0.36, 0.57)	0.004 (-0.14, 0.15)
3-month	41	0.65 (0.55, 0.75)	0.62 (0.52, 0.73)	0.02 (-0.13, 0.17)
4-month	41	0.72 (0.62, 0.83)	0.68 (0.58, 0.79)	0.04 (-0.11, 0.20)
5-month	32	0.72 (0.61, 0.83)	0.77 (0.66, 0.88)	-0.05 (-0.22, 0.11)
6-month	34	0.68 (0.57, 0.79)	0.83 (0.71, 0.94)	-0.15 (-0.31, 0.02)
9-month	30	0.73 (0.60, 0.85)	0.77 (0.65, 0.88)	-0.04 (-0.21, 0.14)
12-month	40	0.74 (0.63, 0.85)	0.84 (0.73, 0.95)	-0.10 (-0.26, 0.06)
**High Risk Fractures**				
6-week	26	0.38 (0.27, 0.49)	0.36 (0.21, 0.50)	0.02 (-0.15, 0.20)
3-month	22	0.59 (0.47, 0.70)	0.55 (0.40, 0.70)	0.04 (-0.14, 0.22)
4-month	24	0.66 (0.55, 0.78)	0.60 (0.45, 0.75)	0.06 (-0.12, 0.24)
5-month	17	0.68 (0.56, 0.80)	0.71 (0.56, 0.87)	-0.03 (-0.22, 0.16)
6-month	18	0.63 (0.51, 0.75)	0.75 (0.59, 0.91)	-0.13 (-0.32, 0.06)
9-month	16	0.65 (0.51, 0.79)	0.67 (0.52, 0.83)	-0.02 (-0.22, 0.18)
12-month	23	0.70 (0.58, 0.82)	0.78 (0.63, 0.92)	-0.08 (-0.26, 0.11)
**Low Risk Fractures**				
6-week	22	0.58 (0.44, 0.73)	0.60 (0.48, 0.73)	-0.02 (-0.20, 0.16)
3-month	19	0.72 (0.56, 0.87)	0.72 (0.59, 0.85)	-0.003 (-0.19, 0.18)
4-month	17	0.80 (0.63, 0.96)	0.78 (0.65, 0.91)	0.02 (-0.18, 0.21)
5-month	15	0.77 (0.60. 0.94)	0.84 (0.71, 0.97)	-0.08 (-0.28, 0.12)
6-month	16	0.75 (0.58, 0.92)	0.92 (0.79, 1.05)	-0.17 (-0.37, 0.03)
9-month	14	0.82 (0.64, 1.00)	0.89 (0.74, 1.03)	-0.06 (-0.27, 0.15)
12-month	17	0.79 (0.62, 0.96)	0.91 (0.78, 1.05)	-0.12 (-0.32, 0.08)

1. Adjusted for treatment, time, fractures-at-risk, baseline questionnaire score, treatment x time, treatment x fractures-at-risk, and time x fractures-at-risk.

2. Repeated measures of variance analysis found a significant effect of time ($p<0.01$), fractures-at-risk ($p=0.01$), and baseline HUI-III score ($p<0.01$) on HUI-III scores. Our analysis failed to show an effect of treatment x time ($p=0.31$), treatment x fractures-at-risk ($p=0.67$), or time x fractures-at-risk ($p=0.76$) on HUI-III scores.
