# Supplementary material for: Trial to re-evaluate ultrasound in the treatment of tibial fractures (TRUST): a multicenter randomized pilot study
Source: Trials. 2014 Jun 4;15:206. doi: 10.1186/1745-6215-15-206 (PMC4060850; doi:10.1186/1745-6215-15-206)
Supplement: Additional file 3 — Adjusted Mean RUST Scores. Description of data: A comparison of adjusted mean RUST scores for the treatment and control groups, at each follow-up time. [file 1745-6215-15-206-S3.pdf]

Additional file 3: Adjusted Mean RUST Scores [1,2]

	N	Sham Device	LIPUS	Difference
		**Mean (95% CI)**	**Mean (95% CI)**	**Mean (95% CI)**
**All patients**				
6-week	49	5.70 (5.16, 6.24)	5.41 (4.80, 6.03)	0.28 (-0.55, 1.12)
3-month	42	8.41 (7.84, 8.97)	7.74 (7.10, 8.37)	0.67 (-0.20, 1.54)
4-month	23	9.47 (8.81, 10.12)	9.30 (8.58, 10.02)	0.17 (-0.85, 1.19)
5-month	14	9.75 (8.83, 10.68)	9.94 (9.15, 10.73)	-0.19 (-1.47, 1.08)
6-month	25	10.62 (9.87, 11.36)	10.25 (9.52, 10.98)	0.37 (-0.71, 1.44)
9-month	14	10.95 (9.86, 12.04)	11.19 (10.27, 12.10)	-0.24 (-1.72, 1.24)
12-month	14	12.05 (10.73, 13.38)	11.12 (10.05, 12.18)	0.94 (-0.93, 2.81)
**High Risk Fractures**				
6-week	26	5.20 (4.54, 5.87)	5.36 (4.51, 6.22)	-0.16 (-1.22, 0.90)
3-month	23	7.71 (7.04, 8.38)	7.49 (6.61, 8.37)	0.22 (-0.86, 1.31)
4-month	15	8.65 (7.94, 9.36)	8.92 (7.93, 9.92)	-0.28 (-1.47, 0.92)
5-month	10	9.03 (8.20, 9.85)	9.66 (8.57, 10.75)	-0.64 (-2.02, 0.75)
6-month	17	10.03 (9.28, 10.79)	10.11 (9.13, 11.09)	-0.08 (-1.29, 1.14)
9-month	9	10.43 (9.38, 11.48)	11.11 (9.90, 12.32)	-0.68 (-2.23, 0.87)
12-month	10	11.27 (10.25, 12.29)	10.78 (9.26, 12.30)	0.49 (-1.35, 2.34)
**Low Risk Fractures**				
6-week	23	6.46 (5.61, 7.32)	5.49 (4.73, 6.25)	0.97 (-0.14, 2.09)
3-month	19	9.48 (8.57, 10.39)	8.12 (7.35, 8.89)	1.36 (0.21, 2.51)
4-month	8	10.74 (9.62, 11.85)	9.88 (9.02, 10.75)	0.86 (-0.43, 2.14)
5-month	4	10.87 (9.23, 12.52)	10.38 (9.31, 11.44)	0.50 (-1.05, 2.04)
6-month	8	11.52 (10.24, 12.80)	10.47 (9.53, 11.41)	1.05 (-0.32, 2.43)
9-month	5	11.76 (9.99, 13.53)	11.31 (10.11, 12.50)	0.45 (-1.30, 2.20)
12-month	4	13.26 (10.79, 15.74)	11.64 (10.31, 12.97)	1.63 (-0.57, 3.82)

1. Adjusted for treatment, time, fractures-at-risk, treatment x time, treatment x fractures-at-risk, and time x fractures-at-risk.

2. Repeated measures of variance analysis found a significant effect of time ($p<0.01$) and fractures-at-risk ($p=0.01$) on RUST scores. Our analysis failed to show an effect of treatment x time ($p=0.53$), treatment x fractures-at-risk ($p=0.611$, or time x fractures-at-risk ($p=0.69$) on RUST scores.
