# Supplementary material for: Trial to re-evaluate ultrasound in the treatment of tibial fractures (TRUST): a multicenter randomized pilot study
Source: Trials. 2014 Jun 4;15:206. doi: 10.1186/1745-6215-15-206 (PMC4060850; doi:10.1186/1745-6215-15-206)
Supplement: Additional file 4 — Adverse events. Description of data: Adverse events reported in both treatment arms. [file 1745-6215-15-206-S4.pdf]

Additional file 4: Adverse events

Adverse event	Sham Device	LIPUS
**Non-operative**		
pain at the site of screws	1	0
superficial infection	0	3
fracture blisters and boils	0	1
pneumonia	0	1
pneumonia-like symptoms	1	0
possible operation-related infection	0	1
low grade fever	0	1
fracture mal-union	0	2
neurapraxia of the saphenous nerve	0	1
hospitalization for deep vein thrombosis and pulmonary embolism	1	0
**Operative**		
implant removal	1	0
implant exchange	0	0
irrigation & debridement	1	5
bone graft	0	0
pre-operative fasciotomy	0	0
post-operative fasciotomy	0	1
amputation	0	0
soft tissue infection	0	0
screw removal	5	3
screw exchange	0	0
fracture non-union	2	1
fracture mal-union	0	0
implant infection	0	1
superficial infection	0	2
fracture gap	0	0
painful hardware	3	1
open wound	0	6
broken nail	0	0
broken screw	0	0
pre-operative compartment syndrome	1	1
post-operative compartment syndrome	0	1
nail migration	0	0
screw migration	0	1
elective screw removal	1	0
